# Pediatric leukemia: origins, pathogenesis, the role of microenvironment and immunological modulation

**DOI:** 10.3389/fimmu.2026.1778415

**Published:** 2026-07-31

**Authors:** Weronika Małkowska, Anna Małkowska, Łukasz Kiraga

**Affiliations:** 1 Faculty of Veterinary Medicine, Warsaw University of Life Sciences, Warsaw, Poland; 2 Department of Toxicology and Food Science, Faculty of Pharmacy, Medical University of Warsaw, Warsaw, Poland; 3 Division of Pharmacology and Toxicology, Department of Preclinical Sciences, Institute of Veterinary Medicine, Warsaw University of Life Sciences, Warsaw, Poland; 4 Military Institute of Hygiene and Epidemiology, Warsaw, Poland

**Keywords:** cancer, hematopoiesis, immunomodulation, microenvironment, pediatric leukemia, inflammation

## Abstract

Leukemia remains the most commonly diagnosed pediatric cancer worldwide. Over the last decade, advances in therapy have significantly improved survival rates; however, treatment outcomes are still unsatisfactory for many patients. Further progress requires a comprehensive understanding of disease biology, including the hematopoietic process, mechanisms of leukemia initiation and progression, the genetic mutations involved, and the cell types that constitute the pre−leukemic and leukemic niches and interact with their microenvironment. Particular attention should be paid to the role of inflammation and immune modulation in the development of this childhood malignancy. Hematopoiesis occurs in three waves, and several hematopoietic niches are involved in cell development and maturation. In these locations, primary pre−leukaemic clones arise prenatally, yet only a subset of cases progress to overt leukemia after birth. In most patients, malignant progression is associated with the acquisition of secondary mutations, which may result from inflammation or exposure to specific infectious agents. Microenvironmental factors play a critical role in this process. Cross−talk between niche components and emerging leukaemic cells gradually shapes a microenvironment that favors malignancy. Extensive remodeling of this niche is driven by structural alterations, deregulation of signaling pathways essential for proper cell function and disturbances in the secretion of immunological factors, thereby creating a supportive and protective environment for leukemia progression. A detailed understanding of fetal hematopoiesis in the context of leukemia, and of how immunomodulatory factors and inflammatory mediators influence disease development, is crucial for improving therapeutic efficacy. This review provides an in−depth overview of these phenomena and, in addition to core information on pediatric leukemia, summarizes recent advances in elucidating its origin and pathogenesis, as well as emerging diagnostic and therapeutic approaches.

## 1 Introduction

**Figure 1:**
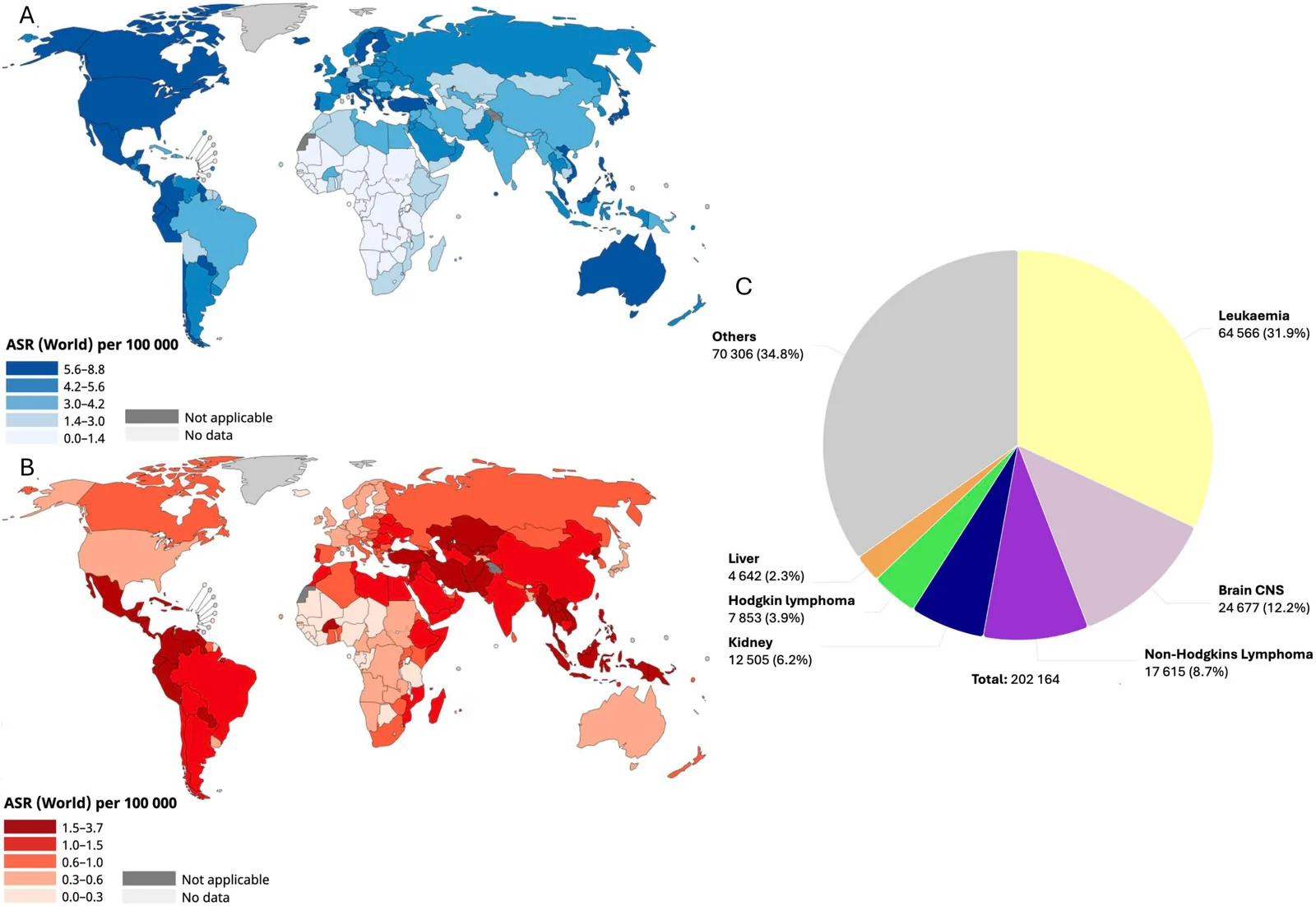
Cancer prevalence and mortality in children. **(A)** Incidence of leukemia in children aged 0–14 per 100–000 in 2022 worldwide. **(B)** Leukemia−related mortality in children aged 0–14 per 100–000 in 2022 worldwide. **(C)** Cancer incidence in children aged 0–14 in 2022 worldwide. Produced with Cancer TODAY (379).

Leukemia remains the most common pediatric cancer and one of the leading causes of death among children under the age of 15 (1, 2). Leukemia still accounts for approximately a third of all cancers diagnosed in children in European countries, including France, Germany and the United Kingdom (1, 3). The data on pediatric leukemia prevalence and mortality rates is presented in **Figure 1**. There are four main types of leukemia, two of which are acute: the most common, acute lymphoid leukemia (ALL), and the less prevalent acute myeloid leukemia (AML). Chronic forms are rare in children and more common in adults, including chronic myelogenous leukemia (CML) and extremely rare chronic lymphoid leukemia (CLL). In addition, juvenile myelomonocytic leukemia (JMML) is a form of myeloid leukemia, but it does not grow as fast as AML neither as slowly as CML leukemia types, and is most prevalent in children under 4 years old (4). In pediatric patients, the most commonly detected mutations include ETV6-RUNX1 and hyperdiploidy, while in infants, KMT2A rearrangements are diagnosed in approximately 80% of ALL patients (5, 6). The most common translocation detected in these patients, typically associated with poor survival, is t(4;11)(q21;q23), known as MLL-AF4 or KMT2A-AFF1 (6–8).

The term “leukemia” was first used in the mid-XIX century by Virchow, who further distinguished splenic and lymphatic types of this disease (9). However, earlier physicians described post-mortem changes associated with leukemia, observed disturbances in blood composition in patients and reported leucocytosis (9). Later, Biermer, schooled by Virchow, reported the first pediatric leukemia patient in 1860 (10). Twenty years later, a report of concordant leukemia in identical twins sparked further observation of other twin pairs and, consequently, helped define the main direction of research on pediatric leukemia development and progression (11). In 1962, a model where leukemia transmission occurs *in utero* between twins via shared circulation was described by Wolman; however, it was not received well among scientists (11, 12). Following this, MacMahon and Levy proposed that leukemia originates prenatally; their study involved collecting and analyzing data from leukemic twins (13). Currently, it is established that leukemia may arise from abnormalities in hematopoiesis at its early stage, in lymphoid-primed multipotent progenitors (14). However, research on the distinguishing specific cell of origin is still ongoing.

The two-hit leukemia model was proposed to describe leukemia development in pediatric patients, according to which leukemia initiation, that is - primary leukemic hit, occurs *in utero* and results in a pre-leukaemic state. The second hit occurs after birth, when secondary mutations are usually acquired, leading to the development of full-blown leukemia (15). Therefore, in children, the first leukemic hit induces the formation of silent, pre-leukemic clones in asymptomatic patients, while the second transformation enhances acute leukemia in children (16). Importantly, in this process, inflammation or infection appears to be a key regulator that can promote leukemogenesis (15, 17).

Importantly, the hematopoietic microenvironment is critical for the generation and maintenance of primary pre-leukaemic clones, as well as their transformation. Moreover, cross-talk between leukemic and niche cells leads to the conversion of the niche into a hospitable and protective microenvironment for the progression of malignancy (16).

This article provides a brief overview of the process of hematopoiesis, then describes the development of leukemia and the most common mutations associated with it. Subsequently, it presents the involvement of cellular niches in leukemia development and their role in the maintenance of malignancy. Next, it discusses the deregulation of immunological factors in this pathology and the impact of inflammatory modulators. Finally, it summarizes current findings that contribute to the understanding of this disease’s development and outlines further research directions, which may lead to improved diagnostic and therapeutic efficacy for this often-fatal childhood disease.

## 2 Hematopoiesis

The leukemia−related research could not have been conducted without primary discoveries in hematopoiesis, which are mentioned in the section below. A profound understanding of this process is essential for further studies on the origin of pre-leukemic cells and their subsequent progression, as well as for facilitating the treatment of various hematological disorders, including malignancies and autoimmune diseases (18). Among them, childhood leukemia is characterized by a high incidence and high mortality rate, especially in infants (1, 6). Further sections explain the process of hematopoiesis and describe hematopoietic niches.

### 2.1 History and background

The discovery of hematopoietic stem cells (HSCs) and their further regenerative potential has its origins in studies on genetics and immunology, mostly associated with transplantology-related investigations (18). The first studies in cattle were based on twin models and focused on the shared circulatory system during the prenatal period; this was followed by attempts at skin transplantation in cattle, and later by studies in mice, in which donor cells were administered intra-embryonically to recipient mice. These studies were of fundamental importance to research on the hematopoietic system and its significance in medicine (18–21). Thereafter, events of the Second World War significantly enhanced research aimed at rescuing damaged hematopoietic systems. This was linked to the discovery of bone marrow (BM) sensitivity to radiation and its impact on subsequent hematopoietic disorders (18, 22, 23). As part of the efforts to restore this system, numerous studies were conducted, including intravenous BM transplants in mice and guinea pigs, followed by an evaluation of methods used in humans, which led to the first evidence of hematopoietic system regeneration (22, 24–26). Consequently, the use of blood stem cells to restore the hematopoietic system following various diseases, including hematologic cancers, has quickly garnered widespread interest among scientists and healthcare professionals (27–35).

At the same time, the mechanism of hematopoiesis has been thoroughly studied. It should be noted that both early research on blood stem cells and subsequent studies, including those currently underway, often use a chicken model. It is considered that Sabin was the leading scientist engaged in investigating the formation of the yolk sac (YS) blood islands. This research was followed by a study by Murray in 1932, who introduced the concept of the hemangioblast formed in an intrinsic manner from primitive streak mesenchymal cells, leading to the formation of hematopoietic and endothelial cell lines (22, 36, 37).

**Figure 2:**
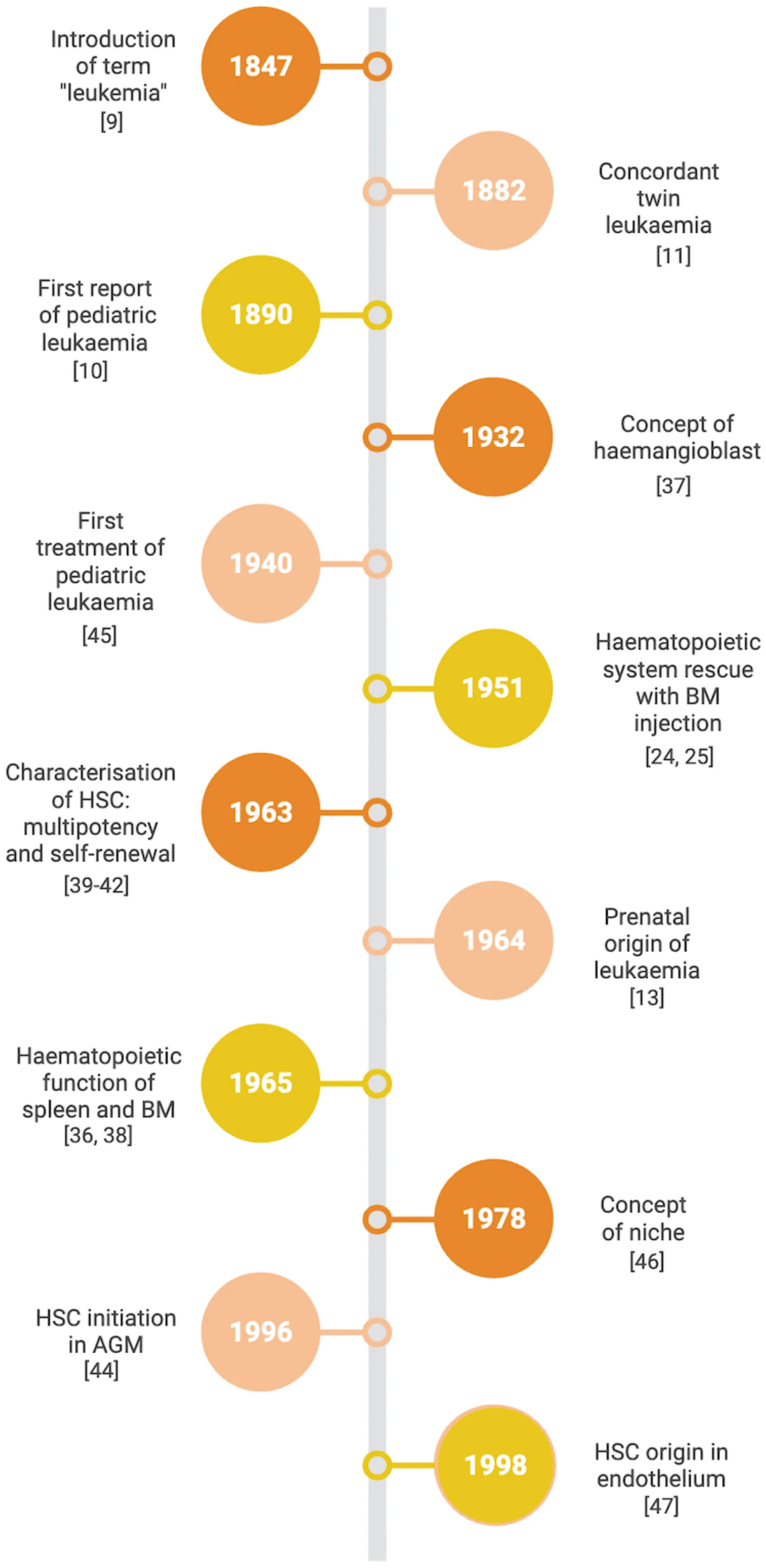
A timeline of the most important discoveries in the field of hematopoiesis and leukemia research prior to 2000. Created with BioRender. Accessed on 16 April 2026.

Subsequently, investigations regarding HSC migration were conducted. It was discovered that the spleen and the BM are populated by blood-producing cells and therefore they both have hematopoietic functions (36, 38). Finally, HSCs were characterized as multipotent cells with self-renewal potential (39–42). The work of Dieterlen-Lievre in 1975 demonstrated that HSCs must have an intraembryonic, not YS, origin (36, 43). Finally, it was discovered that HSCs emerge in the aorta-gonads and mesonephros (AGM) region (44). These studies have laid a solid foundation for further investigation, and since the beginning of the 21st century, important research has been conducted on blood stem cells and, consequently, on leukemia. This has resulted in numerous articles being published each year, and significant ones will be discussed in the following chapters. Crucial discoveries in leukemia -associated research are summarized in **Figure 2** (9–11, 13, 24, 25, 36–42, 44–47).

### 2.2 Hematopoietic stem cells development

Hematopoiesis is a multistage process that occurs in many waves and niches. After immature HSCs arise from the AGM region, subsequent niches, including the spleen, liver and BM, are involved in their maturation and retain hematopoietic potential both in the fetus and postnatally. Hematopoiesis concerns erythrocytes, sustaining the embryo’s oxygen supply and undifferentiated HSCs that will be responsible for hematopoiesis in adult life. Regarding their development process, hematopoiesis can be divided into three main distinctive phases (or waves): the primary - primitive, the secondary - transient definitive (or pro-definitive), and the third - definitive. The first two are HSC-independent, whereas the last wave is HSC-forming (22, 48).

The primitive wave appears in extraembryonic tissues, specifically in the YS, and here the first primitive blood-generating cells are developed, including erythroblasts, erythroid cells, megakaryocytes (implicated in platelet production), and subsequent macrophage lineages involved in the development of lymphatic system and tissue remodeling (22, 48–54).

In the transient wave, key emerging hematopoietic elements include yolk sac-derived myeloid progenitors and lympho-myeloid progenitors (48). This wave appears not only in the YS but also in the embryo, leading to enhanced red blood cell production and establishing the first immune cells (48, 55).

**Figure 3:**
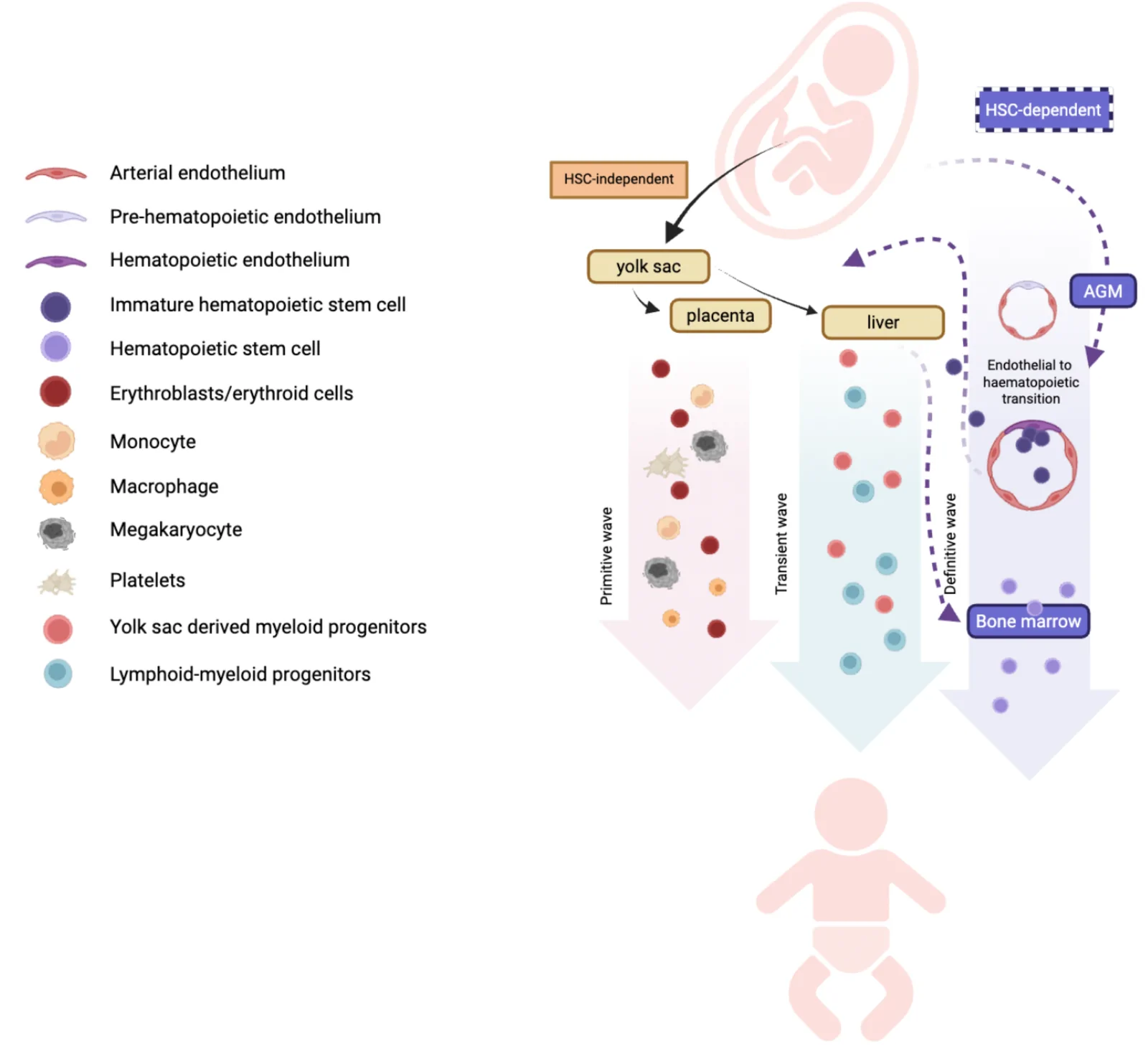
HSC-independent and HSC-dependent waves of hematopoiesis. The scheme illustrates the complexity of subsequent niches where hematopoiesis occurs. Two primary waves have their primary origin in the yolk sac, and progenitors travel mainly towards placenta in primitive wave or liver in transient wave, where their maturation and differentiation occurs. HSCs develop in the last definitive wave, arising from arterial endothelial cells on the ventral side of the dorsal aorta at AGM region, in the process of endothelial-to-hematopoietic transition. Thereafter, immature HSCs travel through the specific niches and finally develop into mature in the liver and bone marrow. In these locations, they become quiescent. Created with BioRender. Accessed on 30 December 2025.

In the last hematopoietic wave, HSCs are generated. Immature cells first appear in clusters in the AGM region in the process of endothelial-to-hematopoietic transition (EHT), specifically on the ventral side of the dorsal aorta (44, 47, 48, 56–58). As EHT initiates in the arterial endothelium, pre-hemogenic endothelial cells first develop. They are further transformed into hemogenic endothelial cells (HE) and subsequently into HSC (48, 59). As reported by Zeng et al., the cells undergoing EHT can be divided into the following categories: endothelial - characterized by CDH5 expression, arterial - expressing GJA5, SOX17, DLL4 and HEY2 markers, and finally - those with hemogenic and hematopoietic characteristics, expressing RUNX1, CD44, MYB, ANGPT1 and SPN, PTPRC, HLF, GFI1B, respectively (60). The arising HSCs can be distinguished from progenitors based on markers RUNX1, HOXA9, MLLT3, MECOM, and HLF, together with SPINK2 (59). Extensive analysis of their markers was performed by Calvanese et al. (48, 59) and it is presented in **Table 1**. EHT is regulated by various transcription factors, including RUNX1 and Gata2 (61). In addition, surrounding tissues influence hematopoiesis: while the dorsal tissues of the embryo suppress the activity of blood stem cells, the ventral tissues promote their development via the Hedgehog signaling pathway (62). The involvement of catecholamines and overall sympathetic nervous system activity in HSC development in the AGM was emphasized by Fitch et al.; the authors observed that Gata3 deletion resulted in disturbance of AGM hematopoiesis (63, 64). Subsequently, HSCs shift into the fetal liver (FL) which appeared to be a crucial site of their proliferation and a base for subsequent expansion (22, 65). Furthermore, during FL maturation, the PROM1/CD133 and HLA-DR markers are acquired by HSCs (59). Multilineage progenitors further develop, and subsequently BM colonization takes place (48, 59). This final migration of HSC from FL to BM is followed by their homing and quiescence (66–68). In summary, during the prenatal period, HSCs are generated in the AGM, proliferate in the FL, and settle in the BM in adulthood (44, 47, 69, 70). HSC-independent and HSC-dependent waves of hematopoiesis are presented in **Figure 3**.

**Table 1:** Characterization of HSC and their precursors.

HSC origin and maturation			
	AGM HSC precursors		
	Arterial endothelium	Pre-HE	HE
Positive markers	HOXA9, MEIS2, SOX17	HOXA9, MEIS2, SOX17, IL33, ALDH1A1, DKK1	RUNX1, HOXA9, MEIS2, SOX17, ALDH1A1, DKK1
Characteristics	Cells of endothelium in AGM, derived from mesoderm.	Precursors of hemogenic endothelium, enable EHT.	Site of EHT and HSC emergence. This transition is orchestrated by RUNX1 and Gata2.
Ref	(44, 48, 59, 380)	(48, 59)	(44, 47, 48, 56–59, 61)
	HSC		
	AGM, YS, Placenta	Liver	BM
Positive markers	RUNX1, MLLT3, HOXA9, SPINK2, HLF, MECOM, LIN288, IGFBP2, HOXB9	After 8^th^ week: RUNX1, MLLT3, HOXA9, SPINK2, HLF, MECOM, HEMGN, MSI2	RUNX1, MLLT3, HOXA9, SPINK2, HLF, MECOM, HEMGN, MSI2
Characteristics	Immature HSC travel through subsequent niches.	Primary location of HSC maintenance, expansion and maturation. The niche comprises hepatoblasts, epitheliocytes, macrophages, and stromal components. It is also a potential location of primary leukemic transformation.	Final HSC niche. Location of HSC proliferation and differentiation followed by quiescence. Comprised of endosteal, arteriolar and sinusoidal components. Crucial in leukemia development, progression, and survival, and highly altered in the presence of leukemic cells.
Ref	(44, 47, 48, 56–59, 61, 79, 83)	(48, 59, 70, 71, 85, 91, 191)	(16, 48, 59, 66, 95, 99)

### 2.3 Niches of hematopoietic stem cells

A niche can be defined as a specialized microenvironment that hosts and provides support and guidance for progenitors and stem cells during their development (69, 71, 72). The concept of the niche was first proposed by Schofield, who stated that external signaling originating from the microenvironment (niche) is required as a crucial regulatory mechanism for stem cell activity (46). Niches are not just regions where HSCs reside, but are directly involved in their functional properties (42, 44, 70, 73). The components of the niche include a variety of factors with specified roles, including regulation, maintenance and protection of stem cells, for example in the case of reactive oxygen species (ROS) accumulation associated with stress and DNA damage (71, 72, 74). HSC niches evolve and undergo restructuring as they age (75). Cheng stated that niches are crucial for preserving the five key characteristics of HSCs, including self-renewal potential, maturation, apoptosis, rest and trafficking (76). In fetal development, crucial niches involved in HSC maintenance and maturation include the liver, spleen, and BM (48, 77, 78). In addition, the placenta is also considered as a HSC niche (79).

#### 2.3.1 Placenta

It was proven that the placenta serves as one of the primary niches for emerging HSC, which occurs simultaneously with AGM. It can also prevent premature HSC differentiation and enable their *de novo* production, especially due to the RUNX1 transcription factor, crucial in hematopoiesis (80, 81). RUNX1 is expressed in both the AGM and the placenta regions (82, 83). The presence of HSCs and progenitor cells was confirmed in the human placenta, and it was demonstrated that hematopoiesis can be sustained by placenta-derived stromal cells (79).

#### 2.3.2 Liver

FL develops from the hepatic diverticulum. It primarily appears in a three-week-old embryo, while the enhanced proliferation of progenitors and subsequent differentiation into hepatocytes is promoted by WNT/β-catenin signaling between the 5th and 10th week of gestation (84). FL consists of a variety of elements, including stromal components such as hepatic stellate cells, endothelium, mesenchymal stem cells (MSCs), fibroblasts, hepatoblasts, epitheliocytes and immune cells, including macrophages (85–90). All these elements create a distinct microenvironment where hematopoiesis and cell differentiation are strictly controlled. FL is required for HSCs maintenance and expansion, and here proliferation radically increases (65, 70). As HSCs migrate into FL, they interact with a new niche formed of hepatoblasts, stromal components, vascular endothelium and importantly – pericytes (89, 91, 92). It was shown that pericytes are engaged in HSC maintenance and expansion, while hepatoblasts regulate erythropoiesis, more specifically – the differentiation and maturation of erythroid cells, via erythropoietin cytokine secretion (89, 91, 92). Furthermore, the stromal stem cell factor (SCF)+ and DLK+ cell population appears to be a crucial factor in HSC expansion due to the expression of various growth factors, including angiopoietin-like 3, insulin-like growth factor 2, SCF, and thrombopoietin (71, 89, 90). Additionally, Zhao et al. discovered that activating transcription factor 4 (AFT4) is essential in further HSC expansion due to upregulation of angiopoietin-like 3 factor and various cytokines (93). In addition, Tamplin et al. designed a study using zebrafish to examine HSC and progenitor cells, their migration, and interactions with the perivascular niche, corresponding to murine FL. The endothelial cell-associated remodeling of the niche into a pocket-like structure, promoted by lycorine, occurs as a result of HSC and progenitor cell colonization and attachment to the mesenchymal stromal component of the niche (94).

#### 2.3.3 Spleen

The next stages of HSC development include their migration from FL to the spleen and subsequently to the BM. These locations contain biologically important HSC niches shortly after birth, especially in the BM, while in the spleen their activity will be more limited (71). It was described by Bertrand et al. that as HSCs colonize the fetal spleen, their proliferation is inhibited, multipotency is lost, and the cells differentiate only into macrophages which secrete anti-inflammatory factors. The spleen contributes to hematopoiesis for up to two weeks after birth (77).

#### 2.3.4 Bone marrow

The BM environment is characterized by specific conditions that promote the proliferation of blood stem cells from birth until the 3rd week of the newborn’s life, after which a state of quiescence sets in during the fourth week (66). Therefore, BM appears to be a crucial niche in controlling HSC proliferation, differentiation, and quiescence, thereby maintaining balanced hematopoiesis (95–98). Among the BM microenvironment, three niches can be distinguished: endosteal, arteriolar, and sinusoidal. They are occupied by a variety of stem and progenitor cell subpopulations, and many non-cellular factors regulate their proper functioning (96–102).

The endosteal niche is comprised mainly of osteoblasts and osteoclasts on the inner bone surface (95, 96). Dominating elements are early lymphoid progenitors, and approximately 15% of HSCs are found here (99, 103, 104). CXC ligand 12 (CXCL12) secreted by osteoblasts only promotes early lymphoid progenitors, and CXCL12-associated maintenance is provided due to its high expression by endothelial cells (103). Subsequently, osteopontin and erythropoietin, osteoblast-derived factors, are secreted, which modulate HSC maintenance and erythroid cell differentiation (95, 105–108).

The arteriolar niche comprises a microenvironment composed of different populations of stromal cells, mostly including CXCL12-abundant reticular cells (CAR) (which are considered a specialized subpopulation of mesenchymal stromal cells) (99, 109, 110) and neural-glial antigen 2 (NG2) positive periarteriolar cells - the crucial elements responsible for HSC maintenance and differentiation or quiescence in the BM, respectively (97, 99). Within this niche, endothelial cells, as well as the nerves of the sympathetic nervous system, and unmyelinated Schwann cells have also been distinguished (95). Due to the low permeability of blood vessels in this location, ROS formation is limited (99, 111).

Sinusoidal niche, on the other hand, is characterized by higher permeability compared to the arteriolar, which favors the activation of HSCs (99, 111). Compared to the arteriolar niche, it lacks supporting cells and contains only a single-layered endothelium (112). Kiel et al. claim that even 60% of HSC occupy this niche (104). In this region, HSCs align closely with CAR cells, which are located around sinusoidal endothelial cells (98). CAR cells are critical components of the microenvironment as they exhibit increased expression of CXCL12 and several other crucial factors maintaining HSCs within the niche (98, 113–115).

##### 2.3.4.1 Cellular component of the BM niche

The mesenchymal stromal cells play an undeniably important role in the BM’s microenvironment (101, 116, 117). Cells with characteristics reminiscent of the mesenchymal stromal cells were first identified in the BM stroma of the guinea pig and described in the literature in 1970 by Friedenstein et al. (118). They were later further characterized and named by Caplan in 1991 as the MSCs (119). Initially, the terms “mesenchymal stromal cells” and “MSCs” were used interchangeably, and sometimes both terms were even used concurrently in the literature as “mesenchymal stem cells/mesenchymal stromal cells” to refer to cells with specific characteristics. In 2005 Mesenchymal Stromal Cell Committee of the International Society for Cell & Gene Therapy (ISCT MSC) issued a paper in which they stated that the term “MSCs” is neither synonymous nor interchangeable with the term “mesenchymal stromal cells” (120). The following year, they published another position paper outlining the criteria for defining mesenchymal stromal cells; according to the definition provided there, a mesenchymal stromal cell should meet the following three criteria: 1) being plastic-adherent; 2) expression and lack of expression of specific hematopoietic and endothelial markers; and 3) capability of *in vitro* differentiation into adipocytes, chondroblasts, and osteoblasts (121). CD90, CD105, and CD73 are considered positive markers, and they lack expression of CD11b, CD14, CD19, CD34, CD45, CD79a, and HLA-DR (100, 116, 121). Mesenchymal stromal cells/MSCs are distributed throughout post-natal tissues; They are most commonly isolated from umbilical cord (blood and Wharton’s jelly), BM, and adipose tissue, but it is known that other sources for their isolation can also include dental pulp, muscle, growth plates, and periosteum (116). Morphologically, they are fibroblast-like cells (122). In general, mesenchymal stromal cells are characterized by their notable secretory, immunomodulatory, and homing properties. They can secrete a variety of anti-inflammatory, immunomodulatory, and angiogenic mediators, enabling them to maintain their niche or support tissue regeneration to which they migrate (116, 117). In the niche, they secrete various factors, including CXCL12 and SCF (100, 123), which support proper HSC development and the regulation of immune responses and inflammatory signaling therein (95, 100). The term “mesenchymal stromal cells” should refer to the general population of multipotent mesenchymal cells, which consists of a minor population of MSCs with precisely defined properties reflecting their true stem “nature” - progenitor self-renewal ability and multilineage differentiation potential (116, 120). However, despite functional differences, MSCs cannot be distinguished yet from mesenchymal stromal cells based on surface markers (116). Thus, MSCs are indistinguishable subpopulation of mesenchymal stromal cells, which have several crucial features: since they are multipotent, when adequately stimulated, they are capable of symmetrical or non-symmetrical divisions into subsequent stem cells or differentiated ones: osteoblasts, chondroblasts, adipocytes, myoblasts (100, 121, 124), and even, under specific *in vitro* conditions – tenocytes (125, 126); however, certain studies prove, that they may also exhibit neurotrophic activity (127) and can induce angiogenesis in infarcted heart (128). Finally, they have significant self-renewal capacity (86, 123).

It appeared that several subpopulations of mesenchymal stromal cells can be identified in the BM niche. Among them, CAR cells, a specialized subpopulation of the mesenchymal stromal cells, appear to be a key HSC maintenance factor (98, 110). Importantly, in the niche, HSCs are located close to CAR cells. Furthermore, they are also involved in the governing of macrophages in the BM niche (101). CAR cells are characterized by increased expression of CXCL12, SCF, forkhead box C1, and early B-cell factor 3 (EBF3), all of which are crucial for HSC preservation (129). Notably, CAR cells isolated from patients with chronic myeloid leukaemia had reduced levels of these factors compared with healthy individuals (129). Greenbaum et al. showed that CXCL12 deletion in this population altered HSC number and their quiescence (130). CAR cells have the potential to further specialize into adipocytes or osteoblasts (129). In 2020, Baccin et al. identified two subpopulations of CAR cells, now termed adipo-CAR cells and osteo-CAR cells. While adipo-CAR cells are localized mainly in sinusoidal BM niche and are characterized by enhanced leptin (Lepr+) receptor expression, osterix-expressing osteo-CAR cells are mainly found in arteriolar BM niches and exhibit low Lepr expression (131). Another key population involved in the maintenance of the BM microenvironment are nestin+ cells. In 2014, Isern et al. divided mesenchymal stromal cells into two subpopulations and demonstrated that nestin+ cells are key factors to establish the BM niche for HSCs and induce their homing within this location, whereas nestin- ones exhibit mainly osteo- and chondroprogenitor function (86, 123). CD146+ mesenchymal stromal cells are considered to be another multipotent subpopulation. These cells are crucial for HSC maintenance and expansion (132); they are involved in HSC preservation and progression within the microenvironment by producing growth factors and Angiopoietin-1, a key driver of vascular remodeling within the niche (132, 133). In 2013, Pinho et al. distinguished the CD146+ subset, nestin+ cells - PDGFRα+ CD51+, which express high levels of HSC niche genes (134). Baccin et al. also identified a cluster of mesenchymal cells expressing Ng2 and nestin, functioning as perivascular pericytes (131). In addition, Prummel et al. demonstrated that the BM niche undergoes remodeling in an inflammatory environment. As part of their research, a specific subpopulation of inflammatory mesenchymal stromal cells (iMSC) was identified (135).

The other cellular components of the niche, which appear to be important for BM microenvironment functioning, include, most importantly, osteoblasts and endothelial cells (86, 95, 98, 99) with emphasis on specific NG2-positive periarteriolar cells, which are crucial elements responsible for HSC maintenance and differentiation or quiescence in the BM, respectively (97, 99). Megakaryocytes, fibroblasts, macrophages, dendritic cells (DCs) and adipocytes are also involved in HSC maintenance (95, 136–139). HSC quiescence is induced by CXCL4 and TGF-β released by megakaryocytes, while fibroblast growth factor 1 (FGF1) secretion promotes their proliferation (95, 136, 137). Macrophage and DC roles include controlling HSC entry into circulation by modulating sinusoidal permeability (99, 138). Within the sinusoidal niche, neurons of the sympathetic nervous system and unmyelinated Schwann cells have also been distinguished (95).

##### 2.3.4.2 Non-cellular components of the BM niche

Non-cellular components, including physical and chemical factors, also constitute a significant part of the niche. A crucial component is the extracellular matrix (ECM). It consists of over 200 different proteins, including collagens, growth factors, cytokines, and enzymes, which are constantly produced, modified, and released by various cells to regulate their microenvironment; as a result, the ECM is effectively balanced and remodeled (95, 140–142). Within BM locations, a variety of factors and distinct roles in HSC maintenance and hematopoiesis. Important chemokine factors secreted into the niche include CXCL12, SCF, and Foxc1 (98, 113–115). Among them, one of the highest importance is CXCL12, a factor involved in the CXC receptor 4/CXC12 (CXCR4/CXCL12) pathway. The highest expression of CXCL12 chemokine is observed in CAR cells, perivascular stromal cells, endothelium, osteoblasts, and even some hematopoietic cells show its low expression (98, 103). It was proven to be a key factor in HSC quiescence and retention in BM, as well as in defense against oxidative stress (143). The self-renewal and differentiation properties of HSC are also mediated by CXCL10 and CXCR3 (144). Furthermore, FLT3 and thrombopoietin (TPO) are other important factors for HSC development and proliferation. Decker et al. demonstrated that systemic TPO elimination affects HSCs within the BM (145–147). Lastly, oxygenation or hypoxia are the key factors regulating HSC function. Interestingly, Spencer et al. reported that the lowest pO2, thus a high hypoxic state, was found in peri-sinusoidal deep locations, whereas in the endosteum, high levels of pO2 were measured (102).

## 3 Pediatric leukemia

Leukemia is a common cancer among children and a highly fatal disease in infants (1, 6). It encompasses a range of subtypes that differ in prognosis (5). Extensive research on the cell of origin and leukemia development, including the importance of inflammation in this process, is ongoing. Furthermore, a growing body of evidence suggests that the microenvironment plays a significant role in the development of leukemia and its progression (16). The following section discusses crucial aspects of leukemia development and describes the subtypes of this disease.

### 3.1 Leukemia prevalence and mutations

#### 3.1.1 Subtypes of leukemia

Childhood ALL can be divided into subtypes arising most commonly from either B- or T-cell precursors, as well as pro-B MLL fusion ALL (15). Regarding the frequency of childhood ALL subtypes, both ETV6-RUNX1 and hyperdiploidy account for approximately 20% cases. Both can be classified as low-risk B-ALL subtypes. In the high-risk subtypes, the most common is the BCR-ABL1-like subtype, which accounts for 12% cases, followed by the less common KMT2A in 5%, then BCR-ABL1, MEF-2D-rearranged and hypodiploid subtypes. Intermediate-risk B-ALL subtypes include PAX5- and DUX4/ERG-rearranged leukemias, each accounting for 4%, while lower frequencies of TCF3-PBX1, ETV6-RUNX1-like, ZNF384-rearranged and iAMP21 subtypes are recorded. Finally, T-ALL subtypes include TAL1, TLX2, HOXA, TLX1 and TAL2 (5). Although the rate of 5-year survival in pediatric leukemia exceeds 80% in children between 1 and 9 years of age, for infants, hence younger than 1 year old patients, the disease, while very rare, is highly fatal (5-year survival is about 50%) (6, 148). Among these youngest patients, mixed-lineage leukemia has the highest prevalence and thus KMT2A rearrangements are the most common mutations in this group. It appears in infants in 50% of AML and 80% of ALL cases and is associated with poor survival in the case of ALL but not in AML (6, 149). Among all subtypes, MLL-AF4 is most frequently diagnosed in almost 50% of ALL cases in infants, followed by less common MLL-ENL and ALL-AF9 in 22% and 16%, respectively. Regarding AML, among 50% of MLL-induced leukemia, the most common aberrations include MLL-AF10, MLL-AF9, and MLL-ELL detected in 27%, 25%, and 15%, respectively (149, 150). In addition, translocation t(9;11) encoding MLL-AF9 is more common in older infants. Therefore, infant leukemia differs from that of older children, in which MLL mutations are less frequent, as observed in 6% of childhood ALL and 14% of childhood AML (148). Thus, a distinction between childhood ALL and its neonatal form has been proposed (149).

#### 3.1.2 Characteristics of leukemic mutations

Pediatric leukemia is often characterized by hyperdiploidy, ETV6-RUNX1 or MLL mutations (5). While the hyperdiploid subtype arises from abnormal cell division, unmutated ETV6 and KMT2A are required to facilitate hematopoiesis (151–153).

Hyperdiploidy arises in patients mainly due to an abnormal amount of DNA/chromosomes. Hyperdiploid ALL can be divided into classical and non-classical types. In the classical one, the prevalent disturbances include disomy, trisomy or tetrasomy heterozygotes, while non-classical is associated with di- and tetrasomy only (151, 154–159). It has been proposed that these mutations arise when chromosomal segregation is disorganized, which results from the influx of cytoplasmic content derived from other cells undergoing division, leading to disruption of the mitotic spindle of the primary cell (151, 155, 160).

ETV6, known as a DNA-binding inhibitor of transcription, belongs to the ETS transcription factor family (152, 161, 162). It comprises three domains, two of which are the main functional domains: oligomerisation promotor N-terminal PNT and DNA-binding C-terminal ETS domains. They are connected by a central domain or linker (152, 161–163). It is a key regulator of hematopoiesis, crucial for prenatal development. Therefore, ETV6 mutations lead to leukemogenesis (152, 164–168). ETV6-RUNX1 fusion results from t(12;21)(p12;q23) chromosomal rearrangement and is the most common mutation, identified in 25% of pediatric ALL cases. Although there is a promising treatment response, many patients relapse (169–171). ETV6-RUNX1 develops prenatally and establishes a pre-leukemic clone that can convert into ALL after birth in two-hit manner (15, 172–177). Importantly, this mutation is not only characteristic of leukemia – it was found to be involved in other cancer types, including colorectal, duodenal or breast cancer (5). A variety of pathways are subsequently deregulated due to ETV6-RUNX1 fusion. They include alterations in anti-apoptotic, proliferative, self-renewal and cell-survival signaling pathways, such as JAK-STAT5, PI3K/AKT/mTOR, RAC1/STAT3/MYC, and many others, leading to the establishment of a pre-leukemic state or progression to leukemia (172, 178–180).

MLL or lysine methyltransferase 2A (KMT2A) gene is a key factor engaged in hematopoiesis control by the regulation of MEIS1 and HOXA gene clusters. Therefore, mutations in these genes can lead to leukemogenesis (153, 181). Due to KMT action, S-adenosylmethionine methyl groups are transported to the H3 histone tail lysine residues, thereby affecting chromatin formation and gene expression (182). KMT2A mutations can lead to chromatin rearrangement and gene expression disturbances and therefore to the emergence of chromatinopathies (181–183). Importantly, given the low frequency of somatic secondary mutations detected in patients, it was suggested that probably even MLL rearrangements alone can cause leukemia (7, 184, 185).

Firstly, it was shown that MLL-AF4 can be involved in promotion of leukemogenesis, as it results in Pro-B ALL when introduced into CD34 human cells (14, 186). Subsequently, Sinha et al. demonstrated that AML can be induced by MLL-ENL recombination in an embryonic mouse model, which occurred during FL hematopoiesis stage. Importantly, when compared to the adult AML model, the pediatric one appears to be of a more aggressive phenotype (14, 187).

Furthermore, it was shown that MLL-AF9 cells can induce leukemia development. In the study on mice by Chen et al., MLL-AF9 from FL and adult BM-derived HSCs were transplanted and as a result, B-cell leukemia and AML developed, respectively. However, after the transplantation of FL cells, the leukemia development was prolonged compared to BM HSC transplantation (14, 188). Lastly, Lopez et al. research conducted on a mouse model showed that megakaryoblastic leukemia development can be associated with fetal HSC ETO-GLIS2 expression. However, compared to the adult BM HSCs, it was shown that ETO-GLIS2 results in rather delayed transformation of a myeloid subtype (189).

Other mutations found in infant leukemia cases are associated with RAS and FLT3 modifications. It has been shown that tyrosine kinase (TK)/PI3K/RAS-activating mutations are present in almost 50% of MLL-induced ALL cases in infants (184, 190). While FLT3-internal tandem duplication (ITD) (juxta-membrane domain) is more common in older children, in infants, FLT3-TK domain (TKD) mutations are more prevalent. FLT3 is a crucial hematopoietic factor and is expressed in early HSC progenitors. Upon ligand binding, the receptor undergoes homodimerization, thereby activating downstream signaling. It includes enhancement of RAS and PI3K pathways, which are crucial for cell development. Therefore, the modifications of this TK, either in the juxta-membrane domain or TKD, can impair hematopoiesis and are associated with leukemogenesis (190).

### 3.2 Leukemia development

#### 3.2.1 Cell of origin

In pediatric patients, the most commonly diagnosed leukemia is ALL of B-cell precursor origin, while the T-cell subtype is associated with approximately 15% of cases (5, 17). Additionally, in infants, mainly pro-B cell subtypes are diagnosed (17). Regarding the development of pre-leukemic clones, Barrett et al. showed in mouse embryo models that MLL-AF4 is associated with three major events occurring between embryonic days 12 and 14 and might induce a pre-leukemic state. They include enhancement of B lymphopoiesis, pre-B cells expansion and increase of blood SC self-renewal potential (191). These changes, together with subsequent disturbances of ETV6-RUNX1 in IL-7R B cell progenitors, appear to be leukemia-inducing. Therefore, described mutations which lead to pre-leukemia origin might appear in early B progenitors, that is, in a population of lymphoid primed multipotent progenitors (14, 191, 192). Importantly, in other studies, BCR-ABL and MLL-AF4 mutations were found in early lymphoid progenitors CD34+/CD19- (193).

Since CD34+/CD19- progenitors showed absence of ETV6-RUNX1 mutation, it was hypothesized and subsequently confirmed that more mature lymphoid progenitors CD34+/CD19+ are the site of ETV6-RUNX1 origin (193, 194). A recent study of O’Byrne et al. showed the presence of similar transcriptomic signatures in early fetal 10-PrePro-B normal progenitor cells and in infant leukemic blasts (91, 195). In addition, the study of Castor et al. proved that the same leukemic mutation can originate from distinct cells. While isoform p210, the major BCR-ABL1 fusion breakpoint, develops in HSC, it is in the B-cell progenitor that the minor breakpoint BCR-ABL1, thus the P190 form, arises. This second (p190) form is associated with pediatric ALL (17, 194). Investigation of leukemia origin in early stages of embryonic development concerned the research on MSC and HSC common precursor. Involvement of pre-hematopoietic precursors in the development of leukemic mutations, including MLL- AF4, BCR-ABL1, ETV6-RUNX1, MLL-ENL, and MLL-AF-9, was evaluated in the study of Menendez et al. In all examined BM samples derived from pediatric patients diagnosed with ALL and carrying these mutations, only MLL-AF4 was confirmed in MSCs (196). In other study, MLL-r, ETV6-RUNX1 and E2A-PBX were found in MSCs of ALL children with corresponding mutations (197, 198).

#### 3.2.2 Two-hit model of leukemia

The two-hit model describes the development of leukemia. While the first hit leads to the prenatal creation of a pre-leukemic state, the second hit involves the acquisition of further mutations, which is associated with inflammation and infections occurring after birth (15). Initial mutations that lead to the ALL pre-leukemic state establishment can involve pro-B cell or B cell precursor subtypes, mainly in infants and older patients, respectively (17). They include ETV6-RUNX1 t(12;21) translocation, TCF-PBX1 t(1;19) translocation, BCR-ABL1 t(9;22) translocation or MLL rearrangements that arise *in utero* (17, 177, 199–201). Thereafter, postnatal secondary events, including further mutation acquisition, can be associated with dysfunction or impaired regulation of immune response, and subsequently, clonal expansion. Regarding MLL rearrangements, as lower incidences of secondary mutations are detected, it is hypothesized that it is sufficient to trigger leukemogenesis (7, 183, 184, 202). In other cases, secondary mutations involving rearrangement of the MLL gene may be necessary for the development of leukemia (17). Additionally, Uckun et al. in 1998 reported a case of a healthy child who was a carrier of a KMT2A rearrangement (17, 203).

**Figure 4:**
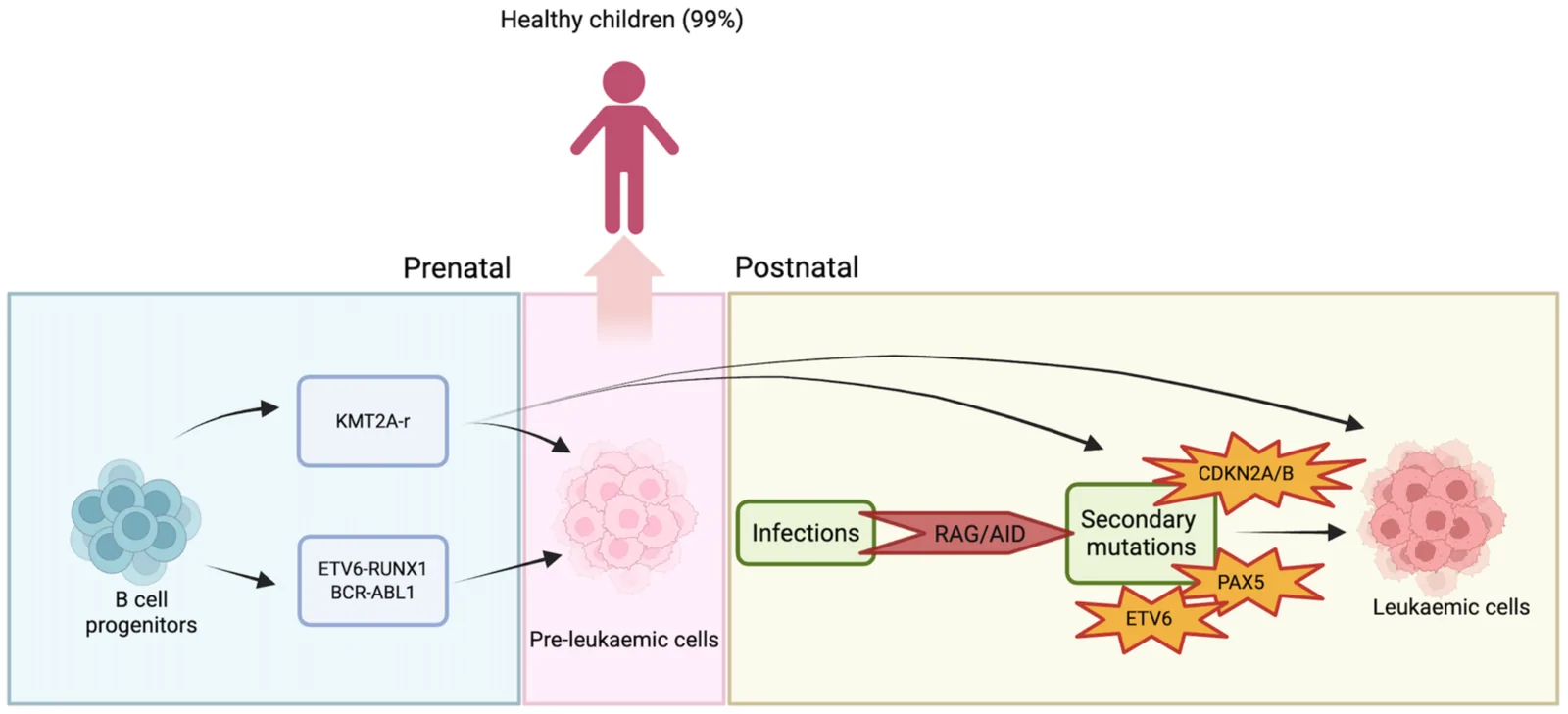
Origin and development of pediatric leukemia. Leukemogenesis occurs in a two-hit manner, with primary pre-leukemic clones arising prenatally from progenitor cells due to mutations such as ETV6-RUNX1 or BCR-ABL1. Thereafter, infections and deregulation of immune system can lead to secondary leukemic mutations in CDKN2A/B, ETV6, PAX5, which drives leukemia progression leading to the development of leukemic cells. KMT2A mutations might be sufficient to trigger leukemogenesis. Created with BioRender. Accessed on 16 April 2026.

Secondary leukemic hits are associated with alteration of important physiological signaling pathways and regulatory mechanisms, thus, tumor-suppression factors and regulators of the cell cycle or apoptosis can be altered (7). They include enhanced activation of RAS or JAK-STAT pathways but also interfere with transcription factors, for example, PAX or IKZF1 and appear as deletions of leukemia-suppressing genes, such as CDKN2A/B (16, 204, 205). Sun et al. described events or mutations that can follow ETV6-RUNX1, including ETV6 deletion, RUNX1 gain, PAX5, leading to leukemogenesis and ALL development (172). Regarding PAX5, it is a key factor in the development of B cells, as it promotes B cell commitment via CD19/CD79a expression and repress certain genes including Flt3, Ccl3 and Notch (206–208). Alterations of this genes including deletions or rearrangements can lead to leukemogenesis (5). The origin and development of pediatric leukemia are illustrated in **Figure 4**.

Fuller et al. developed an innovative screening tool that allows detection of translocations in chromosomes - “genomic inverse PCR for exploration of ligated breakpoints” (GIPFEL) (209). Surprisingly, the ETV6-RUNX1 mutation was found to be carried by 5% of all GIPFEL-screened cord blood samples from healthy neonates (210). However, it was further estimated that leukemia development might occur in 1% of those predisposed children (210, 211).

#### 3.2.3 Importance of inflammation and immune modulation in leukemia development

Infections, inflammations, and dysregulation in the immune system can impact pre-leukemic cells and promote their transformation into leukemia. An extensive analysis of Greaves presents the relationship between the altered immunological response to inflammatory stimuli and leukemogenesis (15).

There are two theories regarding the described issue. The first, proposed by Kinlen, implies that leukemia incidence is strongly correlated with “population mixing” resulting from migration. Second one, introduced by Greaves, is the “delayed infection” hypothesis, which posits that if infants are protected from the exposures to microbes early in life, this later results in highly dysregulated immunological reactions to pathogens (15, 212–214). According to Greaves, although these two approaches to leukemia development appear different, they share a common underlying mechanism: an immune-related disturbance. This is an important paradox of the modern world: diminished infection rates in children resulting from improved hygiene can negatively impact immune system evolution, adaptation, and proper responses to various injurious agents, and, as a result, ALL incidence increases in developed countries (15).

The link between immune disturbances and leukemia development was further evaluated in scientific research. Rodriguez-Hernández et al. showed that ETV6-RUNX1-expressing mice developed B cell precursor of ALL only after exposure to infectious agents; it was associated with mutations in lysine demethylase genes and led to H3K4 demethylase level increase together with RAG2 (215). Furthermore, Martin-Lorenzo et al. demonstrated that Pax5 heterozygous mice develop B-cell ALL precursor only as a result of exposure to pathogens, representing postnatal infections. Leukemogenesis was induced by JAK3 somatic mutations (216). Furthermore, Auer et al. in their recent study confirmed a correlation between PAX5 suppression and upregulation of MYC in leukemogenesis (217). Lastly, T cells and NK cells are also potential investigatory targets as leukemia -supporting cells (17). Therefore, it can be stated that inflammatory factors play a crucial role in HSC regulation and can therefore induce hematological malignancies transformation (15, 214, 218). Papaemmanuil et al. demonstrated that the leukemogenesis of ETV6-RUNX1 pre-leukemic cells is frequently promoted by RAG-mediated mutations. It was shown that in pre-leukemic cells, enhanced activation-induced cytidine deaminase (AID) expression, resulting from aggregation of inflammatory cytokines, especially TGFβ, might be a crucial factor that triggers this process (15, 176). Henceforth, while it was proposed that inflammation might upregulate AID in pre-leukemic cells, which in turn further induce secondary mutations leading to ALL development, Rodriguez-Hernandez et al. demonstrated on mouse models that AID might not be the only leukemogenesis driver (219).

Lastly, the emerging issue which may be related to postnatal leukemia development induced by infections is associated with the gut microbiome and antibiotics. It was discovered that in leukemia -predisposed mice (carrying ETV6-RUNX1 or are PAX heterozygous) the composition of the gut microbiota can be altered. Furthermore, it was shown that infectious stimuli are not required to induce leukemia in predisposed mice whose gut microbiota is altered by antibiotic treatment (211). Recently, Chen et al. conducted an extensive analysis of the gut microbiome and its connection to leukemia, and confirmed that specific bacteria may be involved in leukemogenesis (220). Galland et al.’s meta-analysis shows a major decrease in gut microbiota alpha-diversity in leukemic patients compared to healthy individuals. The most significant changes among bacterial species abundance include increase in *Enterococcus* and *Bacteroides* and decreases in *Lactobacillus*, *Bifidobacterium*, and *Prevotella* (221). Importantly, the gut microbiota have a strong influence on the immunological system by regulatory T cells (Tregs) differentiation and cytokine secretion, such as IL-6 or TNFα. When it is in balance, PRR/TLR signaling maintains a limited inflammatory response towards gut bacteria and activates the immune system in the presence of pathogens. Therefore, in the case of an imbalance in microbial species, the immunological response will be upregulated, leading to chronic inflammation and an enhanced release of proinflammatory factors. In addition, when the intestinal barrier supported by these microorganisms is compromised, LPS and other endotoxins may subsequently enter circulation, leading to systemic inflammation and further promoting the leukemia development (222). Importantly, Kameri et al. considered several factors, including contact with animals, vaccinations, and, in case of newborns, vaginal delivery, skin-to-skin contact and breastfeeding for a minimum 6 months, which can significantly impact children’s microbiome composition, stimulate their immune system and eventually contribute to reducing the risk of leukemia development (223). This can be supported by taking probiotic supplements, particularly when taken by mothers during the final months of pregnancy, as well as by giving them to infants after birth (222).

### 3.3 Leukemic cells in the microenvironment

#### 3.3.1 Pre-leukemic niche

The microenvironment plays an important role in leukemia development supporting early pre-leukemic clones. They further modify the secretions and structures of cellular components, and promote angiogenesis (224, 225). The subsequent establishment of an inflammatory environment enhances leukemia progression. As a result, a malignancy-favoring niche is created, which will promote leukemogenesis and, thereafter, support and protect leukemic cells (99, 226–228).

The importance of the microenvironment in the development of leukemia was confirmed in a study by Wei et al. It was shown that CD34+ cells can undergo malignant transformation into myeloid, lymphoid, but also mixed-linage leukemic subtypes due to MLL-AF9 mutation. Importantly, it appears that microenvironmental factors play a crucial role in favoring the subtypes of multipotent leukemic stem cells (229). Delahaye et al., in their recent study of pre-leukemic cells oncogenesis, demonstrated that within the microenvironment, GAL1 plays a pivotal role in malignant transformation. Notably, GAL1 blockage appears to inhibit leukemogenesis in predisposed cells, which correlates with enhanced IL-7R signaling (230).

Pre-leukemic cells carrying ETV6-RUNX1 or KMT2A can remodel their niche and transform it into a favorable microenvironment by altering adhesive properties, structural components of cells, and key cellular signaling pathways (224, 231). One of the most extensively investigated mutation lesions is the ETV6-RUNX1 mutation, and cells harboring this fusion interact with the niche in an altered manner (224, 231). It is associated with increased expression of CD44, CD18, CD11a, and CD54, as well as decreased expression of CD29, which together enhance adherence of pre-leukemic cells to the endothelium (224). Alterations in cytoskeleton-regulating genes, including those involved in cell dynamics and shape, lead to disorganization of actin and microtubules (99, 224). Impaired CXCL12/CXCR4-dependent migration has also been observed in ETV6-RUNX1 cells (99, 224). In MLL-r cells, increased expression of genes associated with homophilic adhesion has been reported, and abnormalities in apoptotic signaling have also been observed (99, 231). In addition, adhesion properties disturbances were described in the case of BCR-ABL1, which can lead to pre-leukemic niche alterations (99, 194, 201, 232).

Regarding the preleukemic AML niche, Goda et al. recently reported that the presence of preleukemic cells induces substantial alterations (233). A decrease in Lepr+ mesenchymal stromal cells was confirmed; these cells can differentiate into osteolineage cells, including osteoblasts and osteoprogenitors, but also into adipocytes and chondrocytes (233). Vascular remodeling associated with a reduced number of endothelial cells was also observed. In contrast, an increase in pericytes and in certain BM cells that secrete ECM was reported, together with an expansion of CD55+ fibroblasts that promote BM niche remodeling and contribute to leukemogenesis (233). Due to oncogenic signaling and inflammation-induced stress in the microenvironment, oncogene-induced senescence can be activated, driving the transformation of the niche into a leukemia-supporting microenvironment (225, 234). This process was previously described as an anti-tumorigenic mechanism; however, it has also been shown to promote oncogenesis (225, 234). Secretion of vascular endothelial growth factor (VEGF), IL-8, and IL-6 leads to niche modification by promoting angiogenesis and endothelial cell proliferation, and also affects surrounding immune cells (99, 225, 234). Pathways including p16INK4A/Rb and others are stimulated by injurious factors and contribute to these changes (99, 225, 234).

Thereafter, the established inflammatory state further impacts leukemic cells and their niche. It was demonstrated that secretion of TGB-β and, importantly, activin A by mesenchymal stromal cells during inflammation is crucial for the creation of a leukemia-promoting microenvironment (99, 226). Primarily, activin A modulates the niche since it acts as a suppressant of CXCL12 secretion by mesenchymal stromal cells. Secondly, in the presence of activin A, proliferation of ETV6-RUNX1 cells is not affected in the same manner as B cell progenitors, therefore it appears that activin A/TGB-β promote expansion of these pre-leukemic cell clones (99, 226, 235). A similar effect was observed with other pro-inflammatory factors, IL-6, TNFα and IL-1β. Furthermore, in the presence of these factors, ETV6-RUNX1 cells’ migration towards mesenchymal stromal cells via CXCR2 is enhanced when compared to normal cells. Lastly, it was proposed that while inflammation-induced DNA damage occurs in all cells, it can promote further leukemogenesis of more resistant ETV6-RUNX1 clones, especially due to highly expressed AID in these cells (99, 228). These results were confirmed by Whitehead et al., who conducted a study on investigating cytokine levels at birth. High levels of IL-1β, IL-8, TNFα and VEGF, and decreased levels of IL-10 might be observed in children predisposed to develop leukemia compared to the others (236, 237).

**Figure 5:**
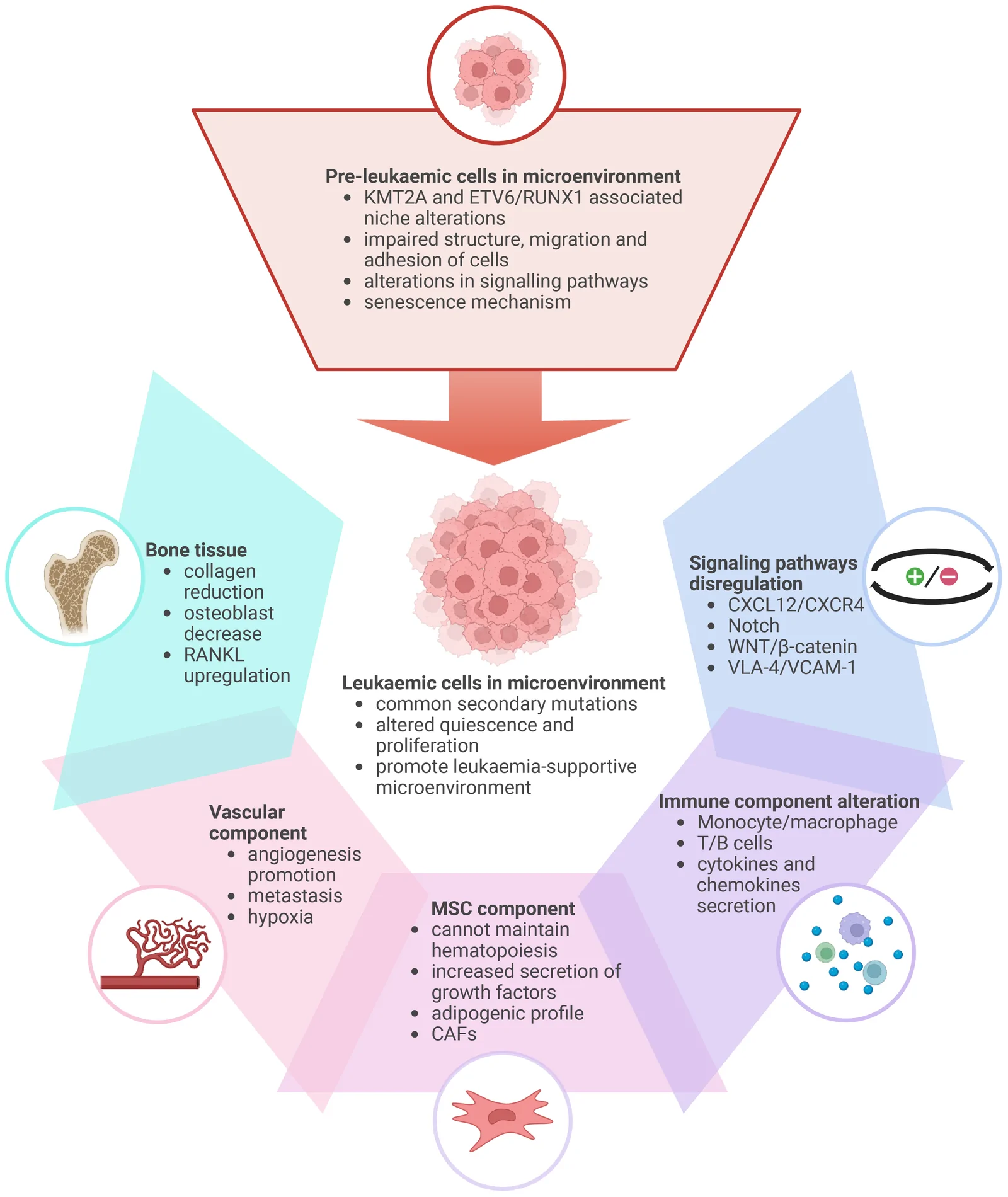
Alterations in microenvironment in leukemia development. Primarily, pre-leukemic cells can alter their niche to a leukemia-favoring state. These cells are often characterized by primary mutations, including KMT2A or ETV6-RUNX1, and may develop into leukemia, often in association with dysregulated immune system or inflammation. The presence of these pre-leukemic cells can induce changes in the structure, adhesion, and migratory properties of the other cells within the occupied niche. Subsequently, leukemic cells can arise within this location. They are characterized by common secondary mutations and deregulation of quiescence and proliferation. As a result of their emergence, the microenvironment further develops into a leukemia-promoting niche. As leukemogenesis progresses, developing malignant cells transform mesenchymal stromal cells, bone cells and vascular elements, leading to the dysregulation of signaling pathways and immune components. As a result, the niche becomes a supportive environment for leukemic cells. Created with BioRender. Accessed on 27 March 2026.

In summary, the microenvironment is crucial for the development of pre-leukemic cells, which can transform their surroundings into a favorable habitat. Inflammation appears to play a pivotal role in this process. **Figure 5** presents an overview of key components of the niche altered during the development of leukemia.

#### 3.3.2 Leukemic niche

As leukemia develops and progresses from pre-leukemic clones, the microenvironment concurrently transforms into a hospitable and protective niche that favors leukemia promotion. Importantly, BM of ALL patients shows elevated levels of HSCs and the presence of very small embryonic-like stem cells (VSELs), which also appear to contribute to leukemia promotion (238). Leukemia maintenance within the niche is associated with the alterations in its structural components, including mesenchymal stromal cells, bone, and vasculature. Mesenchymal stromal cells secretions, such as angiopoietin-1 and SDF-1α, are induced by leukemic cells while bone remodeling occurs with receptor activator of nuclear factor kB ligand (RANKL) enhancement and further angiogenesis promotion (99, 239, 240). Subsequently, crucial pathways for normal cell maintenance, quiescence, differentiation and proliferation or survival in unfavorable conditions, including CXCL12/CXCR4, Notch, WNT/β-catenin and very late antigen-4/vascular cell adhesion molecule (VLA-4/VCAM-1) are deregulated by leukemia presence (239, 241). Finally, immune and inflammation-associated factors such as monocytes and macrophages, T cells and especially Tregs, as well as a variety of cytokines modulate the site of developing malignancy (99, 242–244). As a result, the niche transforms into a leukemia-promoting and treatment-resistant environment.

##### 3.3.2.1 Structural alterations

Three major niche elements crucial for maintaining balanced hematopoiesis, and modified by leukemic cells, include mesenchymal stromal cells, bone cells, and vascular components. As a consequence of their alteration, leukemia maintenance, progression, and survival are enhanced (16, 99). Recently, Ferrao Blanco et al. distinguished mesenchymal stromal cells subpopulations in the BM, identifying mesenchymal progenitors, adipogenic progenitors, and more superficially located osteogenic-lineage cells (245). MSCs are an important niche component that supports HSCs and appears to be profoundly altered by leukemic cells (246). In the study by Conforti et al., mesenchymal stromal cells derived from the BM of leukemia-affected children failed to maintain physiological hematopoiesis to the same extent as in healthy individuals, and the proliferation rate of ALL-associated mesenchymal stromal cells was decreased. However, their morphology, immunophenotype, and potential to differentiate into adipocytes or osteoblasts were similar to mesenchymal stromal cells from healthy donors, and no leukemic-type translocations were found in mesenchymal stromal cells (99, 197). These observations were further supported by Genitsari et al. and Blanco et al. Secretion of growth factors, angiopoietin-1, SDF-1α, CXCL12, and osteopontin by mesenchymal stromal cells derived from the BM of leukemia patients was reported, which may drive further leukemia-favoring alterations within the microenvironment (240, 245). Lopez et al. demonstrated upregulated production of bone morphogenetic protein 4 (BMP4) in mesenchymal stromal cells from leukemic patients compared to healthy individuals, and higher BMP4 production correlated with decreased mesenchymal stromal cells proliferation (247). Moreover, mesenchymal stromal cells exposed to leukemic cells, especially those with the ETV6-RUNX1 subtype, show increased interferon α/β-related gene expression (248). Leukemic cells also promote an adipogenic profile of the niche and enhance the emergence of mesenchymal stromal cells adipogenic progenitors, which in turn induce secretion of IL-7, SCF, and VCAM-1, thereby supporting ALL cell survival (245). Cancer-associated fibroblasts (CAFs) are another crucial element of the leukemic niche. They can arise through several mechanisms, including activation of resident fibroblasts and transformation of MSCs mediated by TGFβ via SDF-1/CXCR4 signaling (249, 250). Pan et al. showed that MSC/CAFs enhance leukemic cell growth, migration, and invasion, mainly through SDF-1/CXCR4 signaling and subsequent PI3K/AKT pathway upregulation (249, 251).

Importantly, engraftment of leukemic cells in the BM can lead to alterations in the bone structural components and matrix. In ALL pediatric patients, bone growth and strength are often affected, and bone fractures can be observed (99). Cheung et al. conducted an extensive analysis of the B-cell precursor ALL effect on bone tissue development. Primarily, it was shown that hematopoiesis in the BM is disturbed due to ALL cells. Subsequently, reduction in collagen, endothelial cells and osteoblasts was observed, accompanied by increased activation of osteoclasts and loss of bone tissue. Since the RANKL is a key factor mediating myeloid precursor differentiation into osteoclasts, its production was investigated in leukemic cells. Since the authors confirmed high RANKL levels, the underlying mechanism of osteoclast enhancement leading to bone resorption was revealed (252).

Finally, cross-talk between the leukemic and vascular components of the BM niche is critical for three significant reasons. First is associated with the source of oxygen and nutrients for rapidly proliferating and growing cells. Second is leukemia progression and metastasis. Third is the impact of leukemia on vascular cells’ secretions (99, 253). Additional microenvironmental factors further contribute to leukemic progression. Importantly, Balandran et al. highlighted the significance of hypoxia for the maintenance and promotion of leukemia-initiating cells (254). Transformation of tumor microenvironment and shift towards dominance of more aggressive leukemia cell populations in response to hypoxia is particularly featured by hypoxia-inducible factors (HIF). HIF is an important factor inducing leukemia progression, survival and importantly, and is involved in remodeling of the vascular niche (239). Furthermore, HIF-1α expression has been previously related to malignant transformation and the promotion of progression (255). It is involved in angiogenesis, regulates HSC quiescence, proliferation and differentiation of cells. Importantly, it is involved in ROS regulation by HSC (239, 255, 256). Since vascular niche alterations include angiogenesis enhancement in the BM of leukemic patients, Wellman et al. conducted research which confirms HIF-1α overexpression in two-thirds of ALL pediatric patients, as well as co-expression of VEGF; an angiogenesis promoter, was detected in half of those HIF-1α positive patients (239, 257). Therefore, HIF appears to be a crucial factor in leukemic cell maintenance and is implicated in treatment resistance determining in patients (239). It was demonstrated that in hypoxic conditions, mTOR/HIF-1α block can improve chemosensitization in leukemic patients (239, 258). Finally, secretion of chemokines, including CCL2 and CX3CL1, by vascular cells can be promoted by the presence of leukemic cells in this niche (99). Thus, the vascular component is essential for maintaining leukemic cells and promoting their progression and invasion; however, it enhances chemoresistance and contributes highly to the leukemia-supportive microenvironment (239). Therefore, all described niche components can be profoundly altered in ways that serve leukemic cells.

##### 3.3.2.2 Deregulation of signaling pathways

Deregulation of signaling pathways is a further consequence of leukemic cells’ development in the BM niche. It concerns the pathways crucial for normal cell maintenance, quiescence, and proliferation, but also their survival. The most important alterations are associated with CXCL12/CXCR4 and Notch, which are highly involved in cell retention and modulation of migration properties within the BM, as well as in differentiation and lineage commitment (239). As an example, WNT/β-catenin and VLA-4/VCAM-1 promote cell growth, adhesion, and survival. Therefore, as a result of these pathways deregulation, leukemic progression and resistance to treatment are enhanced (239, 241).

Two primary pathways that are crucial to sustain normal progression of hematopoiesis and are altered in the leukemic microenvironment are CXCL12/CXCR4 and Notch. They are involved in early embryological development, including cell commitment and regulation of this process, proliferation, survival, but also organogenesis (239). CXCR4/CXCL12 pathway is an important migration-guidance and quiescence factor in the BM niche (99, 259). In ALL patients, this pathway is dysregulated; while CXCR4 expression is enhanced, production of CXCL12 is decreased (99, 259). The underlying mechanism is associated with the previously mentioned, but sustained in the microenvironment, and concerns activin A expression in the leukemic BM niche, which reduces secretion of CXCL12 by mesenchymal stromal cells. Importantly, it appears that in an environment enriched in activin A, migration of leukemic cells towards CXCL12 is more efficient than that of healthy (99, 226). Other BM-derived chemokines which production is enhanced in ALL patients include CCL2, CXCL1 and IL-8 (99). Polak et al. demonstrated that the leukemic niche can be further altered: CXCL10, IL-8 and CCL2, survival-enhancing cytokines, can be produced at increased levels via leukemia-induced tunneling nanotubes that stimulate mesenchymal stromal cells to secrete these factors (99, 260). However, apart from the CXCL12/CXCR4 axis, the role of CXCL12/CXCR7 in establishing a leukemia-promoting microenvironment is also being investigated. It is associated with the function of CXCR7 in early hematopoiesis in the developing embryo and the regulation of HSC migration properties and homing in the BM (261–263). CXCR7 has been shown to play a key role in tumor development of other cancer types, including breast and lung cancer, as it promotes cell adhesion, growth, and survival; it also appears to be a potential factor in tumourigenesis (262, 264, 265).

The Notch pathway is commonly associated with oncogenesis in T cells in ALL, and Notch1 mutation is present in up to 60% of T-ALL cases. The implication of B-ALL is also evaluated, including the involvement of Notch3/4 in the anti-apoptotic properties of these cells (239, 266–268). Regarding T cells, Notch1 is one of the main regulators of their precursor development, as it suppresses alternative differentiation signaling and promotes T cell commitment (267). Furthermore, when altered, it promotes T-ALL cell progression, engraftment, and maintenance within the BM, leading to subsequent growth, and affects different signaling pathways, including mTOR, while suppressing pro-apoptotic cellular mechanisms (241, 267). On the other hand, in the development of B cells, Notch serves as an inhibitory signaling (267). It appears that Notch signaling can enhance oncogenesis in the case of B/T- ALL. Importantly, Notch3/4 is involved in mesenchymal stromal cells-promoted anti-apoptotic protection and chemoresistance of B-ALL cells (267, 268). It is particularly interesting due to its role in the differentiation of other cells, including components of the BM niche. Hilton et al. demonstrated an important role of Notch-induced suppression of osteoblast differentiation, leading to preservation of mesenchymal progenitors in BM (267, 269).

Therefore, chemokine disturbances and dysregulation of CXCL12/CXCR4 and Notch pathways due to mutations can impair normal hematopoiesis and promote leukemogenesis in the BM microenvironment (239, 241, 270). However, WNT/β-catenin and VLA-4/VCAM-1 signaling pathways are crucial for cell maintenance, survival and enhancement of resistance to treatment (241, 271–274). It was shown that mesenchymal stromal cells can support ALL protection and enhance treatment resistance, and this effect is mediated via activation of WNT signaling, which is important in the regulation of cell proliferation and survival (241, 272). In addition, deletion or downregulation of WNT inhibitors was detected in relapsed patients (275). This signaling pathway was studied to a greater extent in solid cancers, but also in leukemia, where cells require it to maintain self-renewal potential. WNT/β-catenin upregulation was reported in T/B- ALL (271, 272). Another chemoresistance-promoting factor which is also highly involved in cell adhesion is VLA-4. Its overexpression was reported in relapsed patients’ leukemic blasts, but also in hematopoietic cells (99, 241). The drug resistance is mediated by cross-talk between leukemic cells expressing VLA-4 and VCAM-1 on mesenchymal stromal cells in the BM, which promotes nuclear factor (NF-κB) activation, a crucial factor in chemoresistance enhancement (99, 274).

##### 3.3.2.3 Deregulation of immunological factors

The importance of inflammatory secretions in all stages of leukemia development, including pre-leukemic state establishment, was discussed in previous chapters. However, these factors are critical to further shape the leukemia-promoting and protecting niche (99). Key components of the immunological system that are altered in the leukemic microenvironment include monocytes, macrophages, T cells, and various inflammatory mediators and cytokines (99, 243). In addition, extracellular vesicles appear to play a pivotal role in the deregulation of the BM niche and immune modulation, causing leukemia progression, low survival and metastatic properties (276).

Therefore, as leukemia progresses, a variety of factors are deregulated in the microenvironment. Lee et al. demonstrated that monocytes influenced by increased leukemia-promoting production of CXCL10 can further enhance ALL cells migration and invasion potential (99, 277). Furthermore, it was shown that the other chemokines that impact monocytes and macrophages migration, CCL2, CX3CL1 and C5a, appear to be upregulated in the BM of leukemia patients (278). The C5a increase is of particular interest due to its role in polarization of macrophages into the M2 pro-tumorigenic subtype via NF-κB activation and appears to be correlated with PTX downregulation (278, 279). The crucial role of M2 macrophages and their impact on the environment, associated, among others, with enhanced secretion of VEGF, CXCL10, and TGFβ, was extensively investigated, in particular in CLL (280). Additionally, an increase of M2 macrophages was confirmed in BM analysis of pediatric and adult ALL patients, which was proven by Hohtari (99, 281). Valencia et al. demonstrated that ALL-associated overexpression of BMP4 results in the transformation of DC cells into an immunosuppressive type and in the promotion of M2 macrophage polarization (99, 282).

T cells are other crucial elements in the leukemic microenvironment. Wiggers et al., in their recent study, conducted an extensive analysis of pediatric T-ALL microenvironment. They confirmed a disbalance of cell populations; especially increased level of double-negative TCRαβ was observed, but decreased level naïve T cells and NKT cells. In addition, they demonstrated changes in the population of B cells (283). The most important mechanism that enables leukemia maintenance and provides support to evade T cells-associated anti-leukemic immune response includes alterations in immune checkpoint expression and Tregs aggregation (242). There are many immune checkpoints, including cytotoxic T cell antigen 4 (CTLA-4), programmed cell death protein 1 (PD-1), whose expression was shown to be enhanced in CD4 and CD8 T cells derived from the BM of ALL adult patients. In pediatric leukemic patients, T cells were investigated, and CTLA- 4 and PD-1 increased expression was reported (242, 281, 284). Tregs are T cells that express Forkhead box P3 (FoxP3), a crucial factor that controls and modulates inflammation via cytokine secretion, including IL-10 and TGFβ. Tregs subsequently inhibit other pro-inflammatory cells, such as T/B cells, NK cells, and macrophages (242, 244). Their accumulation was confirmed in ALL patients which appears to promote leukemia progression (242). Importantly, while IL-10 (an anti-inflammatory cytokine) deficit might be potentially an important driver of leukemogenesis, its increased levels in patients diagnosed with ALL can inhibit anti-leukemic immunological response (243, 285–288). It is associated with suppressive effects on T and NK cells, pro-inflammatory cytokines release and deregulation of DC antigen presentation (285). Furthermore, Li et al. investigated Helios Tregs in pediatric ALL and demonstrated their abundance. It was shown that they can modulate the BM microenvironment by promoting angiogenesis via the VEGFA/VEGFR2 pathway (99, 289).

Finally, inflammatory mediators play a crucial role in primary leukemogenesis and in maintaining a leukemia-supporting niche. Leukemic patients show elevated levels of several cytokines, including IL-10, IL-6, CXCL-10, and CXCL-9 (286, 287). Dai et al. also reported increased IL-6 and IL-10, together with elevated IFN-gamma and decreased TGFβ in the serum of ALL patients (290). Due to the abundance of inflammatory mediators, multiple signaling pathways critical for cell proliferation, survival, and stress responses become dysregulated (99, 243). In pediatric ALL, hematopoiesis and cell differentiation are disrupted by BM secretion of pro-inflammatory cytokines and growth factors, such as IL-1β, IL-4, IL-2, TNFα, IFNγ and GM-CSF (99, 227). IL-6 is a key cytokine that promotes leukemogenesis and niche modulation by enhancing endothelial cell migration and regulating VEGF-mediated angiogenesis, thereby securing oxygen and nutrient supply for leukemic cells; it also promotes stem-like properties in malignant cells (285). A pro-inflammatory niche rich in TNFα and other cytokines further reshapes the microenvironment. It affects mesenchymal stromal cells and endothelial cells, inducing secretion of chemokines such as CCL2, which alters macrophage migration and polarization (99, 278). Leukemic cells are additionally supported by increased IL-8 and CCL2 production driven by enhanced adhesion between ALL cells and BM-derived MSCs, which improves survival and proliferation of protective MSCs (291). Elevated TNFα levels are associated with a leukemia-promoting state and increased angiogenesis, whereas reduced TNFα has pro-apoptotic effects (285). Moreover, TNFα amplifies inflammation and disease progression via upregulation of NF-kB, PI3K/AKT, and MAPK pathways (243), leading to overexpression of apoptosis-suppressive factors such as Bcl-2 and surviving, also observed in case of IL-6 upregulation (285). NF-kB also additionally contributes to dysregulation of CXCR4/CXCL12 and VLA4/VCAM1 interactions, thereby strengthening ALL cell support within the BM niche (292). G-CSF upregulation promotes mobilization of HSCs and myeloid cells from the BM niche into the peripheral blood through alterations in the CXCL12/CXCR4 axis (259). In T-ALL, IL-7/IL-7R is a crucial regulator of normal lymphopoiesis that activates STAT5, PI3K/AKT, and MEK/ERK pathways; its upregulation enhances these pathways, promotes leukemogenesis, and supports cell survival via Bcl-2 upregulation (293, 294). In addition, recent data indicate that IL-7R mutations can directly induce leukemic transformation in T-ALL models (293, 294). Many other cytokines and soluble factors influence the pediatric leukemia microenvironment, sustain inflammation, and drive leukemic progression through diverse mechanisms and signaling pathways (243). These mediators are summarized in **Table 2** (243).

**Table 2:** Crucial immunomodulatory factors in TME, their mechanisms and impact on leukemic cells.

Factor	Mechanism	Impact on leukaemic cells	Ref
CXCL10	Increased production of CXCL10 due to presence of leukemic cells	• Enhance migration • Promote invasion properties via MMP9 • Monocytes shift into inflammatory profile	(277)
CCL2	Increased production due to stimulation with IL-6, TNFα and IL-1β	• Increase adhesion properties into BM niche • Survival enhancement • Involved in macrophages polarization	(278, 291)
CX3CL1	Upregulated in leukeamic patients	• Potential monocytes recruitment	(278)
C5a	Upregulated in leukemic patients, potentially correlated with PTX3 downregulation	• Potential role in recruitment and M2-polarisation of macrophages via NF-KB	(99, 279)
IL-8	Upregulated in leukemic cells presence	• Promote BM mediated adhesion of leukemic cells • Support survival of niche stromal cells	(291)
CXCL12	Downregulation in presence of leukemic cells	• Crucial to preserve population of healthy cells	(259, 278)
G-CSF	Upregulated in presence of leukemic cells	• HSC/myeloid cells mobilization into blood from BM	(259)
BMP4	Overexpression in leukemic patients	• Profiling DC into immunosuppressive type • Enhancement of M2 macrophages polarization	(282)
IL-6	Increased in leukemic patients leading to upregulation of STAT3, NF-KB, PI3K/AKT and VEGF	• Promote Bcl-2 expression • Enhance endothelial cells migration • Promotion of stem-like properties • Suppress apoptosis • Promote proliferation and survival	(285)
IL-10	Increased in leukemia patients	• Decrease T/NK cells activity • Deregulate DC antigen presentation • Suppress inflammatory factors secretion	(243, 285, 286)
TNFα	Increase leads to leukemia -promoting features while decrease shows pro-apoptotic properties. Can lead to upregulation of STAT3, NF-KB, PI3K/AKT and VEGF	• Promote Bcl-2 expression • Increase of migration and proliferation of endothelial cells • Promote angiogenesis via matrix metalloproteinase expression enhancement • Increase leukemia survival	(285)
IL-7	IL-7/IL-7R signaling alteration and mutation in IL-7R, promote STAT5, PI3K/AKT, MEK/Erk	• Induce leukemogenesis • Bcl-2 upregulation • Leukemic cells promotion and progression	(243, 293, 294)

## 4 Future perspectives in pediatric leukemia: emerging treatments, modeling approaches and artificial intelligence implementation in pediatric patient management

### 4.1 Novel treatments in pediatric leukemia

Advancements in pediatric patient approaches and the incorporation of novel methods into existing protocols have significantly improved therapeutic outcomes and reduced therapy-related toxicity. Novel leukemia treatments include personalized chimeric antigen receptor (CAR)-T cell immunotherapy, antibody-based therapies, including monoclonal antibodies, antibody-drug conjugates and a novel construct, bispecific T cell engager (BiTE) and small molecule inhibitor therapies, such as BCR, ABL1, TKIs, JAK inhibitors, proteasome inhibitors, mTOR inhibitors, FLT3 inhibitors and menin inhibitors (295–302).

One of the novel and very promising approaches of leukemia management is CAR-T immunotherapy. FDA approved the first CAR-T therapy in 2017 for ALL treatment, while the first promising results in adult leukemia patients were achieved in CLL a few years earlier (300, 303, 304). The therapy is based on the isolation of patient T cells, genetically engineering them ex vivo, and their administration. To enhance T lymphocyte binding to tumor cells, genetic engineering has been used to develop chimeric antigen receptors of T cells. These engineered receptors confer specific immune functions to the effector cells. Initially, CAR-T cells were designed to selectively identify surface structures on target (tumor) cells in a manner that does not depend on the major histocompatibility complex (MHC) (305). As a result, upon infusion, CD19-expressing cells can be recognized by CAR-T cells, leading to an anti-tumor effect (306). Since 2017, several other CAR-T therapies have been approved, but only tisagenlecleucel (Kymriah) remains available for pediatric patients (307). However, there are major limitations of CAR-T therapy, including especially CD19- relapses and commonly observed adverse reactions, such as cytokine release syndrome and neurotoxicity (300, 308, 309). Therefore, this method must be further optimized to improve patients’ long-term outcomes and minimize undesirable effects of the therapy. An example of an investigated approach is CD19/CD22 cotransduced CAR-T therapy (310).

Other emerging approaches in pediatric leukemia treatment include antibody-based therapies. Among them, the usage of BiTE appears to be a very promising strategy. Its mechanism of action is based on simultaneous binding of a BiTE to CD19 on leukemic cells and CD3 on T cells, followed by T cell activation, resulting in the effective eradication of ALL cells (311). Blinatumomab was approved for the treatment of pediatric leukemia patients, after its efficacy was confirmed, especially when compared to conventional chemotherapy effectiveness (311–313). Despite significant improvements with this type of therapy, one of the remaining challenges is loss or reduction of CD19 expression in relapsed patients (314). To address this limitation, novel constructs are being developed, such as CD22-targeting BiTE (315). The BiTE construct is further investigated, and there are attempts of its advancements, such as Simultaneous Multiple Interaction T-cell Engaging (SMITE) and checkpoint inhibitory T cell-engaging (CiTE). In general, research on bispecific T cell-engaging antibody is still evolving (316–318).

Among small-molecule inhibitor therapies, the emerging strategy is the menin inhibitor, investigated in KMT2A ALL patients. The significance of menin in KMT2A leukemogenesis was confirmed 20 years ago, and the results of recent clinical trials with revumenib in pediatric and adult patients are very promising (302, 319–321). Novel therapies are critical for the improvement of patient outcomes; however, several challenges still persist, including resistance to therapy, toxicity, relapse and high costs (300, 307–309, 314).

### 4.2 Modeling approaches in childhood leukemia

There are several novel modeling approaches to further improve patient outcomes. One of the well-studied and essential models is the patient-derived xenograft (PDX), where leukemic cells obtained from humans are transplanted, most often into mice. Thanks to that, first - the leukemia development, progression and interactions between leukemic cells and the microenvironment can be studied; second - precise therapies can be developed and evaluated, and third - mechanisms of relapse can be investigated (322–326). Moreover, the use of mice as leukemia models allows for the generation of a humanized niche that enables the study of healthy and leukemic cells’ behavior within the microenvironment and how it can be transformed under their influence (327–330). Importantly, PDX biobanks are being established to improve preclinical research, including studies on cancer biology and determination of disease characteristics before treatment implementation; this approach is also applicable in the evaluation of the efficacy of novel therapies and the assessment of therapy resistance, which may prove to be very beneficial in relapsed patients (331–333). Another PDX model utilizes zebrafish; Azzam et al. conducted a study comparing larval zebrafish PDX models with mouse PDX models of solid tumors from pediatric patients. Results showed that the novel zebrafish PDX might be an alternative to the mouse model, as it is characterized by a more efficient engraftment procedure and lower costs (334). Current research is evaluating the zebrafish as a PDX model to improve the treatment of hematological malignancies and serve as a platform to study pediatric leukemia pathogenesis and niche interactions (335–337). Organoids also appear to be an important model for studying the interactions of cancer cells within their microenvironment; however, although they represent a very promising new platform, further research is needed on their effective use in modeling childhood leukemias (338–342).

### 4.3 Artificial intelligence in leukemia research

It seems that the implementation of artificial intelligence (AI) in pediatric leukemia research is emerging as a key approach. The research focused primarily on stochastic and mathematical modeling of the development of leukemia in children, as well as on optimizing their treatment, to achieve the highest possible efficacy while minimizing side effects (343–345). Nowadays, one of the main AI branches is machine learning (ML). It is a potentially highly effective technology that can be implemented in adult oncology (346). ML was primarily used to estimate ALL and AML pediatric patients response to therapy (347–350), although the risk of adverse reaction incidence was also investigated with it (351, 352). As an example, Xiao et al. used ML to predict the occurrence of tumor lysis syndrome in ALL patients (352). Importantly, ML can be used to predict patients relapse or resistance to treatment (353–357). Furthermore, Cheng et al. demonstrated that ML can be used in early screening tools, designed to identify children potentially susceptible to develop leukemia (358). ML also enables the recognition of high-risk patients based on their microRNA signatures (359), whereas Mahmood et al. used ML to establish correlations between patient characteristics and their environmental factors to identify ALL development risks (360). Importantly, optimization of novel therapies can be significantly improved with ML/AI implementation, and research on potential therapeutic targets in hematological malignancies can also be strengthened using these tools (361–363). ML was used to determine genes that might serve as prognostic factors but also significant therapeutic markers for the precise therapy of adult patients with AML (364, 365). Several studies focus on drug-sensitivity research which can be significantly advanced with ML (364–367). Although tools applicable in pediatric oncology are rapidly progressing, the use of AI/ML-based techniques in pediatric leukemia management is still limited, and further studies are required to successfully implement them in clinical practice (356, 357, 368).

### 4.4 Future perspectives

Significant improvement in patient treatment is required to enhance further outcomes. There are several limitations and disadvantages concerning the use of novel therapeutic methods, such as CAR-T and BiTE in pediatric patients. They include common relapses in patients, the phenomenon of CD19 downregulation, severe adverse effects, and the high costs (300, 308, 309, 314, 369–371). Ongoing research is aimed at overcoming these limitations. This includes promising studies on alternative constructs, such as CD19/CD22 CAR-T, SMITE and CiTE (310, 316–318). The incorporation of novel methods at earlier stages of treatment is also being evaluated, as was done in a recent trial conducted with blinatumomab (372). Further research is needed to efficiently adapt novel constructs to the treatment of other hematological malignancies, such as T-ALL and AML (370, 373–376). Ongoing research regarding molecular targeted drugs is very promising. The incorporation of novel agents into early treatment protocols could significantly improve outcomes for pediatric patients in the future (302, 321, 377, 378). Also, AI implementation in pediatric leukemia appears to be a very promising approach, which may accelerate forthcoming discoveries.

## 5 Conclusion

A comprehensive understanding of the hematopoietic system is essential for leukemia -oriented research. The process of fetal hematopoiesis, including HSC development and maturation, involves three distinctive waves that occur in several niches, such as the liver, spleen and BM, but also the extraembryonic placenta. It is known that leukemia development occurs in a two-hit manner, as pre-leukemic clones are established *in utero*, while secondary events, associated with infections and inflammation, lead to final cell transformation after birth. In this process, hematopoietic niches are of critical importance - the HSC microenvironment evolves into one that protects leukemic cells and supports leukemia development. Notably, leukemia includes a variety of subtypes diagnosed in pediatric patients, and infants should be considered separately because of distinct mutational differences.

In conclusion, while in recent years understanding of hematopoiesis and leukemia development has greatly improved, there is still a need for further research. Early diagnostics of highly predisposed children is critical to enable the incorporation of the most efficient treatment at the right time, thereby preventing the development of malignancy. Further research regarding the leukemic microenvironment is essential. Profound knowledge on HSC development, their niche, and their implications in leukemogenesis is critical for the development of effective treatment of hematological malignancies in children and infants to enhance their survival. However, thanks to the continuous evolution of novel diagnostic and therapeutic approaches, treatment outcomes are steadily improving.
